# Recurrent Glioblastoma Resection with Microvascular Free Flap Reconstruction and Associated GammaTile Implantation: A Personalized Approach with Oncologic and Reconstructive Integration

**DOI:** 10.1055/a-2576-7559

**Published:** 2025-04-25

**Authors:** Russel T. Wagner, Jacopo Berardinelli, Amin B. Kassam, Julian E. Bailes, Melanie B. Fukui, George C. Bobustuc, Sammy Khalili, Neil S. Mundi

**Affiliations:** 1 Intent Medical Group, Endeavor Health Advanced Neurosciences Institute, Northwest Community Hospital, Arlington Heights, Illinois, United States; 2 Division of Neurosurgery, Department of Neuroscience, Reproductive and Odontostomatological Sciences, University of Naples Federico II, Napoli, Campania, Italy; 3 Endeavor Health Neurosciences, NorthShore Neurological Institute, Evanston, Illinois, United States

**Keywords:** Glioblastoma multiforme, GammaTile therapy, microvascular free flap, Multidisciplinary management, recurrent brain tumor, scalp reconstruction, brachytherapy, oncologic integration, tissue viability

## Abstract

**Background:**

Glioblastoma multiforme (GBM), despite aggressive multimodal treatment comprising surgery followed by chemoradiation, is almost uniformly associated with inevitable recurrence and poor outcomes. In this clinical context, local radiation therapy—an emerging approach—has gained considerable attention over time for its potential to address the limitations of traditional treatment options for GBM. Multiple surgeries and adjuvant chemoradiation therapy can negatively impact the integrity of the scalp soft tissues and can compromise the ability to achieve primary closure over the surgical site. In these circumstances, complex reconstruction with free tissue transfer may be necessary.

**Methods:**

We report the case of a 37-year-old female patient with recurrent GBM and associated wound healing complications who underwent single-stage GammaTile surgically targeted radiation therapy combined with microvascular free flap scalp reconstruction.

**Results:**

Immediate free flap reconstruction over the site of GammaTile implantation did not result in any wound healing complications and did not compromise the viability of the transplanted tissue. This approach also provided immediate and localized radiation, possibly enhancing patient progression-free survival while reducing the likelihood of radiation-induced adverse effects.

**Conclusion:**

We report the first case of GammaTile implantation with immediate reconstruction of the overlaying soft tissue defect with a free flap. Despite the immediate local radiation produced by the tiles abutting the deep surface of the free flap, there were no complications noted in the vascularity of the transplanted tissue. This finding provides preliminary evidence supporting the safety of using free tissue transfer alongside GammaTile implantation for complex reconstruction.

## Introduction

Glioblastoma multiforme (GBM) is the most prevalent malignant primary brain tumor in adults, characterized by an incidence rate of 3.2/100,000. 1 The standard management of GBM involves a multimodal strategy, including neurosurgical resection followed by the administration of concurrent chemoradiation therapy typically initiated 4 to 8 weeks after the initial surgery. 1 2 3 4 Despite these interventions, GBM is notorious for its aggressive local progression and poor prognosis, with tumor recurrence inevitably occurring, especially near the surgical resection cavity or in immediately adjacent regions where the density of microscopic residual tumor cells is highest. Notably, 50 to 70% of glioblastoma patients experience tumor regrowth adjacent to the resection cavity during the critical 4 to 8-week recovery period before postoperative radiation begins. 1

Several clinical trials have explored the impact of increasing radiation doses directed at the treatment cavity on controlling GBM regrowth. However, while these studies showed only marginal improvements in survival, they also demonstrated a significant increase in radiation-induced adverse effects. 5 To augment the radiation dose directed at the resection bed while mitigating adverse effects to adjacent healthy tissue, various brachytherapy approaches have been explored both for primary and recurrent diseases.

GammaTile therapy, or surgically targeted radiation therapy (STaRT), is designed to address the aggressive recurrence of tumors like GBM directly at the source. 2 This Food and Drug Administration (FDA)-licensed device utilizes cesium-131 (Cs-131) seeds encased in a resorbable collagen matrix, forming a tile that is implanted immediately after tumor resection. 3 The cesium-131 isotope is favored due to its relatively short half-life of 9.7 days, compared to 59.4 days for iodine-125, which has also been used in brain brachytherapy. 1 2 4 This shorter half-life allows for a potent dose of radiation to be delivered directly to the tumor bed, reducing the potential for radiation damage to surrounding healthy tissues.

Each GammaTile measures 2 cm × 2 cm and contains four radioactive seeds. This modularity and the pliability of the collagen matrix ensure conformal radiation delivery, making surgical implantation quicker and aiding in precise dosimetric planning. 3 The design includes a tissue offset of 3 mm, which minimizes the risk of focal necrosis around the radioactive sources. GammaTile delivers 120 to 150 Gy at the cavity surface and maintains 60 to 80 Gy up to 5 mm depth, which is 1.5 to 2× the standard dose delivered by external beam radiation therapy (EBRT). 1 2

The implementation of subsequent surgeries as well as standard adjuvant chemoradiation therapy and surgically targeted radiation therapy can negatively impact the integrity of the scalp soft tissues and can compromise the ability to achieve primary closure over the surgical site. In these circumstances, complex reconstruction with free tissue transfer may be necessary.

Free flaps are often utilized in complex reconstructive surgeries, particularly in cases where large defects are created after tumor resection, such as in head and neck cancers or soft tissue sarcomas. 6 7 8 These flaps of well-vascularized tissue are essential in covering defects and promoting healing in previously irradiated or compromised tissues. However, postoperative complications such as wound dehiscence, fistula formation, and flap necrosis can pose a significant risk, especially when combined with postoperative radiation therapy or brachytherapy. 6 7 8 9 Studies have demonstrated complication rates as high as 38.33% in patients undergoing microsurgical free flap reconstruction with intraoperative brachytherapy, with the most frequent issues being wound dehiscence and delayed flap necrosis. 7 9 Additionally, patient-related factors such as smoking, diabetes, high BMI, prolonged operative time, anemia, atherosclerotic calcifications, serum creatinine, and prealbumin levels, and extensive intraoperative resuscitation have been associated with an increased risk of flap failure. 10 11 12 13 14 15 16 17 18 19 Despite these risks, free flaps continue to play a crucial role in improving surgical outcomes by providing adequate coverage for large defects and facilitating healing, particularly when combined with careful perioperative management.

## Case Presentation and Surgical Course

A 37-year-old woman diagnosed with GBM (IDH wild-type, WHO grade 4) initially underwent a right temporal craniotomy and tumor resection. Postoperative pathology confirmed high-grade glioma with vascular proliferation, geographic necrosis, and palisading necrosis, consistent with GBM. Molecular testing also revealed that the tumor was IDH1 negative. The patient underwent postoperative adjuvant chemotherapy and intensity-modulated radiation therapy (IMRT). Three months after completing IMRT, she presented with wound induration which was initially managed with incision and drainage of underlying purulent material. All microbiological examinations, including wound collection cultures, yielded negative results.

**Fig. 1:**
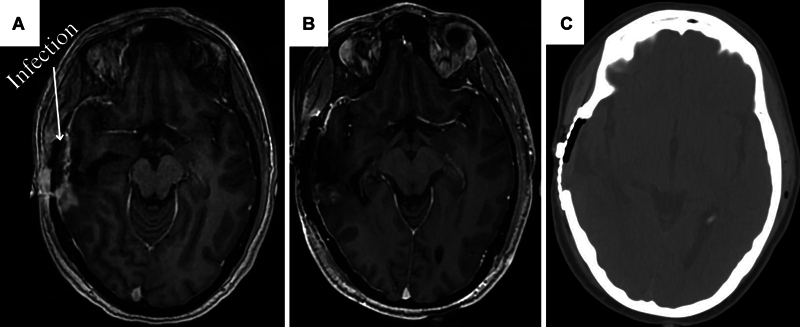
( **A** ) Preoperative axial MRI demonstrating the right temporal GBM, highlighting the extra-axial infection as well as the recurrent tumor's location and extent before surgical intervention. ( **B** ) Postoperative axial MRI. ( **C** ) Postoperative axial CT illustrating the integration of titanium mesh following craniotomy.

The patient continued to experience intermittent yellow drainage and scab formation at the incision site, indicating a persistent wound complication. Blood tests and cultures ruled out systemic infection. At this stage, she was treated conservatively with empiric antibiotics. However, due to the concerns about local infection spread and to rule out the formation of an epidural or intracranial abscess, a follow-up MRI was performed. The imaging showed a small extradural collection sub-adjacent to the craniotomy characterized by restricted diffusion, likely related to the underlying blood product. Also, a postcontrast enhancing nodule in the posterior region of the operative cavity was noted. This finding was neuroradiologically consistent with either tumor recurrence or radionecrosis ( Fig. 1 ).

Given the suspicion of the presence of an epidural infection, approximately 1.5 months after wound incision and drainage, the patient underwent a revision craniotomy. Purulent material was confirmed in the epidural space and after removal of the infected bone flap and debridement of the epidural space collection, a titanium mesh cranioplasty was performed. A temporoparietal fascial flap was used both for hardware coverage and primary scalp closure was achieved. No tumor resection was performed during this procedure. Wound cultures returned positive for Staphylococcus aureus. The patient was then started on a 6-week course of Cefazolin administered via a PICC line, followed by suppressive therapy with oral cefadroxil.

**Fig. 2:**
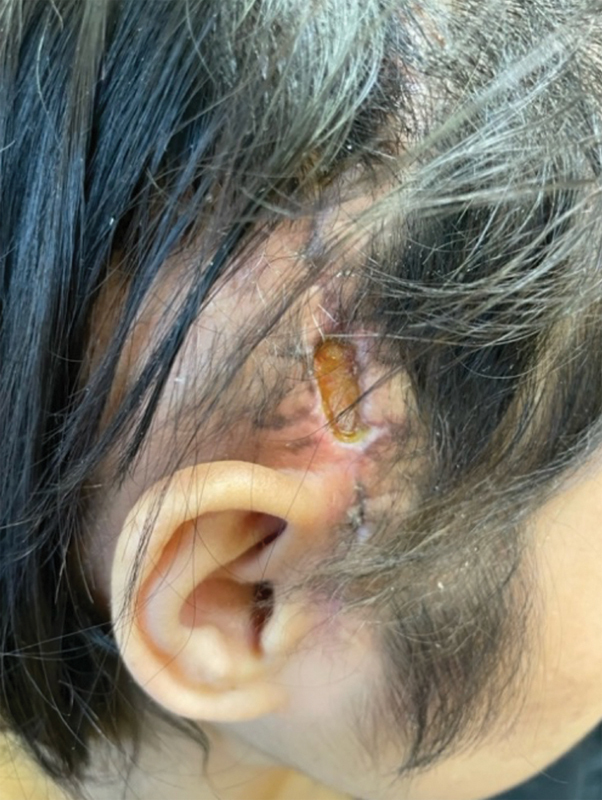
Right temporal wound dehiscence with exposure of underlying temporoparietal flap.

Approximately 3 weeks following surgery, the patient's right temporal scalp skin became dehiscent with exposure of the underlying temporoparietal flap ( Fig. 2 ). A follow-up MRI was performed and did not reveal any sign of residual infection but showed a volumetric progression of the nodular enhancing areas with a mild increase in the extent of heterogeneous enhancement.

**Fig. 3:**
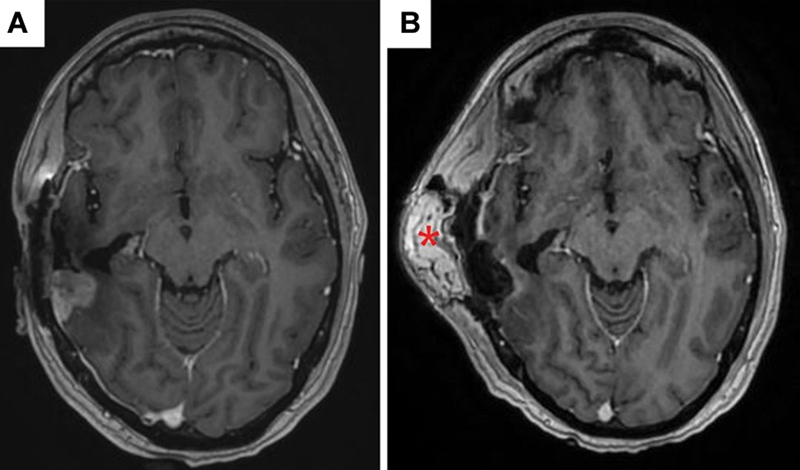
( **A** ) Preoperative MRI showing nodular enhancement in recurrent neoplasm before resection, GammaTile placement, and free flap. ( **B** ) Postoperative MRI after surgery with free flap intact.

In response, a conscious craniotomy was performed for recurrent tumor resection followed by the placement of 3 GammaTiles along the resection bed. Intraoperatively, pathology confirmed viable residual high-grade glioma in the brain surface, midportion, and deeper sections of the right temporal region. Given the poor condition of the patient's temporal scalp skin, she underwent excisional debridement leaving a sizeable full-thickness scalp defect overlying the resection site. To achieve wound closure and dural coverage, a fasciocutaneous anterolateral thigh free flap was harvested and the pedicle was anastomosed to the right facial artery and common facial vein. Fig. 3 demonstrates the pre-and postoperative imaging findings.

**Fig. 4:**
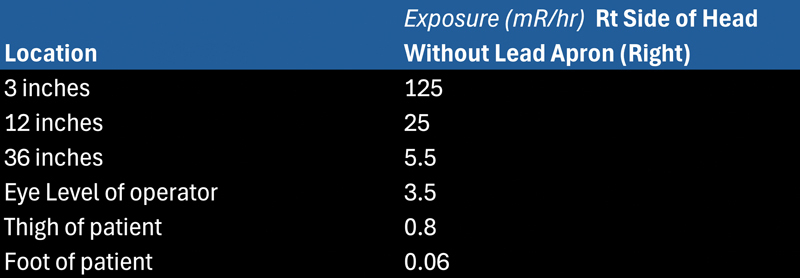
Radiation exposure (mR/hour) at varying distances from the GammaTile.

At the conclusion of the surgery, the radiation physicist assessed radiation exposure at different distances from the surgical site ( Fig. 4 ). The dose measured at the right side of the patient's head, at a distance of 3 inches from the surgical cavity, resulted in 125 mR/hour. This provided the most accurate estimate of the minimal dose the free flap received, though the actual dose to the flap would have been much higher given that the GammaTiles sit closer than 3 inches to the reconstructed tissue. This close proximity increases radiation exposure, raising concerns about the long-term effects on tissue viability. Additionally, the musculocutaneous perforators that perfuse the skin were small in caliber (1–2 mm) and located adjacent to the resection cavity. Despite these challenges, the free flap remained viable in the immediate postoperative period, suggesting that GammaTile therapy can potentially be safely integrated with reconstructive procedures. However, further research is necessary to fully understand the effects of such high, localized radiation doses on free flaps.

## Postoperative Course

Postoperatively the patient had an uneventful course of recovery. She remained in hospital for 7 days and the free flap demonstrated no evidence of arterial ischemia or venous congestion. The incision lines also remained intact and the patient had an excellent neurological outcome with no deficits.

**Fig. 5:**
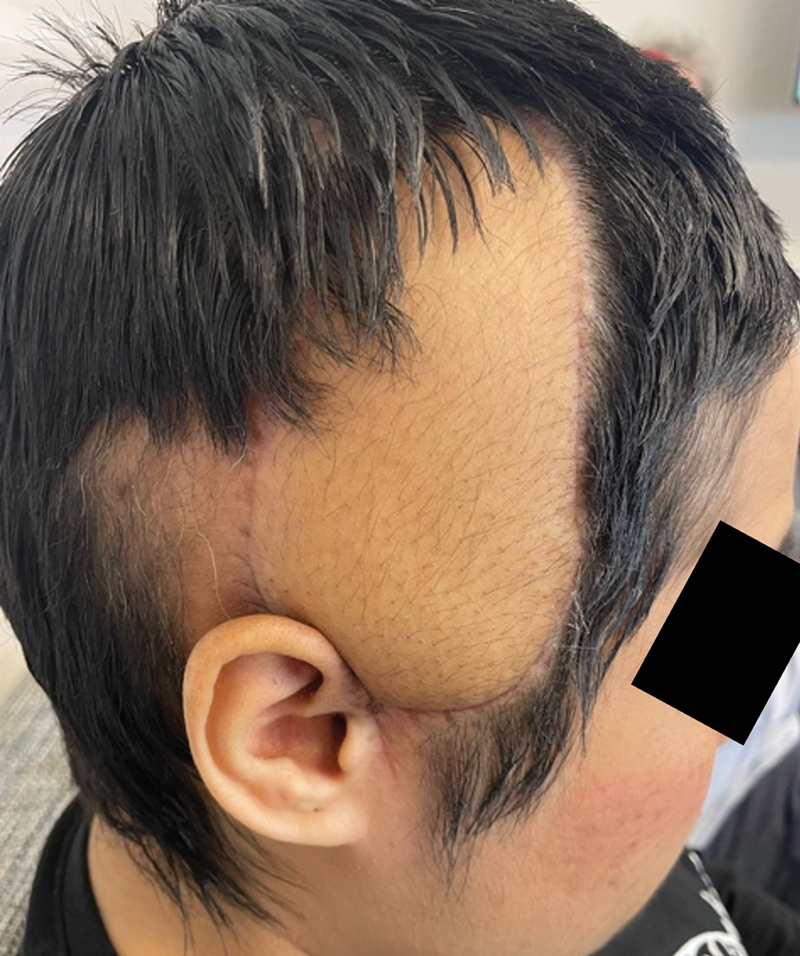
Right scalp free flap reconstruction 9 months following surgery.

At over 9 months from surgery, the free flap has now completely healed, providing soft tissue coverage of the right temporal region with an excellent aesthetic outcome ( Fig. 5 ). This integrated approach of single-stage GammaTile therapy for GBM with free flap reconstruction is the first of its kind reported.

## Discussion

The management of GBM presents ongoing challenges due to its aggressive nature and high likelihood of local recurrence. This case report presents an innovative approach in which GammaTile therapy and free flap scalp reconstruction can be integrated simultaneously to address both oncological and reconstructive needs in GBM treatment. By integrating GammaTile therapy into the surgical procedure, the patient benefits from immediate localized radiation, which is crucial given the rapid proliferation typical of GBM. This method could potentially reduce the window for tumor regrowth that is usually observed during the delay before starting conventional EBRT. 3 GammaTiles also enhance the precise targeting of remnant tumors and reduce radiation exposure to normal brain regions. The customization aspect of placement intraoperatively also presents an advantage in delivering precise, targeted therapy, which is particularly helpful in eloquent brain regions. 2

Brachytherapy has historically been associated with an increased risk of complications, particularly in patients undergoing microvascular free tissue transfers. Traditional brachytherapy can exacerbate the risk of wound dehiscence and fistula formation, crucial considerations in surgeries involving extensive tissue manipulation. 2 6 8 9 Radiation from brachytherapy can also damage the blood vessels that perfuse free flaps, resulting in tissue ischemia and necrosis, further complicating the recovery process. 7 Beyond radiation exposure, factors such as smoking, diabetes, elevated BMI, prolonged operative time, anemia, atherosclerotic calcifications, serum creatinine, and prealbumin levels, and extensive intraoperative resuscitation have been identified as potential contributors to flap failure. 10 11 12 13 14 15 16 17 18 However, the impact of these factors may vary depending on the type of free flap and the surgical context. 19

Given the complexity of this case, alternative reconstructive options were considered. Local rotational flaps, such as the temporoparietal or pericranial flaps, were deemed inadequate due to prior radiation exposure, wound dehiscence, and the need for durable soft tissue coverage over the GammaTiles. Skin grafting was not a viable option as it would not have provided the necessary bulk and vascularity to cover the exposed hardware. A regional island flap using the trapezius was deemed inappropriate for several reasons. First, the location of the defect in the temporal scalp would be difficult to reach with either an upper or lower trapezius island flap unless an atypically long flap was harvested which would put the vascularity of the most distal aspect of the flap (arguably the most crucial for reconstruction) at risk. Second, the trapezius island flaps would result in suboptimal aesthetic outcomes for the patient as they are bulky and must remain attached to their vascular pedicle. 20 Ultimately, an ALT flap was chosen for its reliable vascular supply, sufficient soft tissue bulk, and flexibility in contouring to the defect. Free tissue transfer also allowed for optimal revascularization, reducing the risk of further wound breakdown and necrosis in a previously compromised surgical field. 6 7 8

A multidisciplinary team approach was crucial in developing and executing a comprehensive treatment plan. This patient's care was coordinated through a multidisciplinary tumor board involving neurosurgery, neuro-oncology, neuroradiology, head and neck reconstructive surgery, and radiation oncology. The treatment plan was developed through discussions at a multidisciplinary tumor board to ensure optimal oncologic control and reconstructive feasibility. Collaborative decision-making focused on preoperative imaging, prior radiation effects, and flap selection to minimize complications and promote long-term healing.

The success of this approach can likely be attributed to the specific characteristics of the Cs-131 used in GammaTiles. Cs-131 is a low-energy emitter with a short half-life of 9.7 days, allowing for a quicker and more concentrated delivery of radiation. 1 2 4 This impacts the efficacy and safety of radiation delivery because after placing the tiles into the operative bed, they instantly deliver a uniform radiation dose to the target area. 3 Fifty percent of the therapeutic dose is delivered in the first 10 days after surgery to help deter residual tumor cells from multiplying, and 88% of the therapeutic dose is delivered within 30 days, with over 95% of the dose delivered within 6 weeks. 3 This rapid and focused delivery reduces the duration of radiation exposure and could decrease potential tissue toxicity. Crucially, the highest doses are delivered during the most critical healing period for free flaps, raising concerns about wound complications. Despite this, we noted no issues with flap necrosis, fistula formation, or wound infection in the 9 months following surgery.

## Conclusion

This case report demonstrates a pioneering approach in the treatment of GBM by integrating GammaTile therapy with microvascular free flap reconstruction. This approach highlights the possibility of meeting complex reconstructive needs without forgoing the ability to address the aggressive nature of GBMs, providing immediate, localized radiation to reduce tumor recurrence risks while preserving surrounding healthy tissues. The utilization of Cs-131 in GammaTiles minimizes radiation-induced toxicity, enhancing patient recovery and improving quality of life through better aesthetic and functional outcomes. This novel approach could potentially set new standards for treating GBM and similar cancers, highlighting its significance for future clinical practices and research in oncology.
